# Effect of type 2 diabetes mellitus caveolin-3 K15N mutation on glycometabolism

**DOI:** 10.3892/etm.2019.8307

**Published:** 2019-12-09

**Authors:** Yiyuan Huang, Yufeng Deng, Lina Shang, Lihui Yang, Juanjuan Huang, Jing Ma, Xianshan Liao, Hui Zhou, Jing Xian, Guining Liang, Qin Huang

Exp Ther Med 18: 2531-2539, 2019; DOI: 10.3892/etm.2019.7840

Subsequently to the publication of the above paper, the authors have realized that there were a couple of errors featured in the final sentence of the second paragraph of the Discussion appearing on p. 2537. First, “may be restricted due the K15N mutation” should have been written as “may be restricted due to the K15N mutation”; secondly, “hypoglycemia” should have been written as “hyperglycemia”. Therefore, this sentence should have appeared as follows (changes highlighted in bold): “The IR/PI3K/AKT/GLUT signaling pathway may be restricted due **to** the K15N mutation and may result in glucose accumulation, which is exhibited in **hyperglycemia** and during the development of T2DM (59)”.

The authors regret that these errors were not picked up upon before the article went to print, and apologize to the readership for any confusion these errors may have caused.

